# Dynamic Computation in Visual Thalamocortical Networks

**DOI:** 10.3390/e21050500

**Published:** 2019-05-16

**Authors:** Roy Moyal, Shimon Edelman

**Affiliations:** Department of Psychology, Cornell University, Ithaca, NY 14853, USA

**Keywords:** vision, metastable, oscillations, pulvinar, awareness, coherence, phase, representational spaces

## Abstract

Contemporary neurodynamical frameworks, such as coordination dynamics and winnerless competition, posit that the brain approximates symbolic computation by transitioning between metastable attractive states. This article integrates these accounts with electrophysiological data suggesting that coherent, nested oscillations facilitate information representation and transmission in thalamocortical networks. We review the relationship between criticality, metastability, and representational capacity, outline existing methods for detecting metastable oscillatory patterns in neural time series data, and evaluate plausible spatiotemporal coding schemes based on phase alignment. We then survey the circuitry and the mechanisms underlying the generation of coordinated alpha and gamma rhythms in the primate visual system, with particular emphasis on the pulvinar and its role in biasing visual attention and awareness. To conclude the review, we begin to integrate this perspective with longstanding theories of consciousness and cognition.

## 1. Introduction

Historically, vision has been viewed primarily as a feedforward pipeline [1,2]. In classic models of visual object recognition, the visual system is often reduced to a set of hierarchically organized, unidirectionally connected units, each tuned to a different combination of input patterns [3,4]. While useful in many applied scenarios, the dynamics of feedforward models have little in common with those underlying perception [5,6,7,8]. In awake, behaving animals—and in certain recurrent neural networks (e.g., echo- or liquid-state machines [9,10])—external stimulation often gives rise to ordered sequences of transiently stable population responses [11,12,13,14,15]. While those do not typically correspond to classic point attractors or limit cycles in the underlying state space [16,17] (as in, e.g., Hopfield networks [18]), they may nonetheless contribute to the generation of reproducible patterns on larger spatiotemporal scales [10,13].

In the special case where a near-perfect balance of excitation and inhibition [19,20] in a neural network results in criticality [21,22] (i.e., when it is poised on the edge of chaos [23,24,25]), the system acquires useful computational properties. The critical regime is characterized by long-range correlations [26], enhanced representational capacity [27], power law scaling [28], alternating periods of increased and decreased phase synchronization (at different spatiotemporal scales [29]), and rapid reconfiguration in response to external perturbations [25,28]—the hallmarks of the wakeful brain [30]. In this regime, metastable dynamics—that is, coordinated activity patterns punctuated by rapid transitions [17,31]—are often observed (see Section 2.1).

Although many models and accounts treat cognitive-perceptual processes as sequences of transitions between attractive states [17,32,33] (a term used here as a catch-all, in reference to either classic attractors or periods of enhanced phase coupling spanning parts of a network), the geometric and topological properties of these transients are still poorly understood. In particular, neuroscientists have only recently begun to investigate the correspondence between metastable states in simple models and repetitive wave patterns in large-scale networks [34,35]. The functional significance of oscillatory stabilization, moreover, is still debated: while accounts that invoke coherence [36,37], synchrony [38], and cyclicality [7,8,39] contend that it contributes to complex forms of neural computation (specifically, ones requiring the prolonged maintenance of representations, such as sensory awareness and working memory), some argue that it is not strictly necessary [40,41].

What is the physical structure of sensory representations? By virtue of what mathematical properties are some patterns of neural excitation separable in an intrinsic (observer-independent) sense [42,43,44,45]? This article aims to bring together high-level theories that derive the brain’s self-organizing [46] behavior from first principles [11,12,13,17], low-level accounts that attribute functional roles to coordinated nested oscillations in ensembles of nonlinearly interacting cortical and thalamic neurons [36,37,47,48,49], and central concepts and measures developed as part of longstanding computational theories of consciousness and cognition [42,44,45,50]. To this end, we use the mammalian visual system as a case study and argue that, in routing and sustaining information, it likely utilizes metastable dynamics.

In Section 2, we outline three neurodynamical frameworks—coordination dynamics [17,46,51], winnerless competition [11,13,16], and chaotic itinerancy [52]—and relate them to recent results and perspectives. We then explore the relationship between criticality, metastability, causality, and representational capacity (defined in terms of the repertoire of separable metastable states that the system may visit). Representational content, we propose, could be empirically characterized by studying the geometry and the topology of the network’s collective dynamics in a time interval of interest.

In Section 3, we survey neurophysiological accounts, models, and data that implicate phase alignment, spike-field coherence, and cross-frequency phase–amplitude coupling in coordinating neural information representation and transfer. We survey the mechanisms underlying the emergence of coupled alpha and gamma oscillations in the mammalian visual thalamocortical system and explore their relationship to visual attention and awareness. The contributions of pulvinocortical interactions to the selection and maintenance of visual representations in awake primates are examined in detail. The evidence, we propose, suggests a correspondence between phase-aligned nested oscillations (and spike-field coherence) [49], metastable network states, and visual awareness.

In Section 4, we begin to integrate this unified approach with central ideas from three neurocomputational theories: Integrated Information Theory (IIT) [42], the Global Neuronal Workspace (GNW) framework [50], and the Free Energy Principle [53].

## 2. Neural Computation as a Dynamical Process

Transient shifts in the coupling between neuronal populations have long been thought to underlie perception and cognition. Hebb [54] was the among the first to suggest that distributed thalamocortical ensembles may coordinate their firing and act as a closed system; similar notions were expressed by von der Malsburg and Willshaw [55] and by Damasio [56], who proposed that complex neural representations are carried by time-locked activity in distributed populations [57]. Others stressed the functional significance of reentry—the recursive exchange of information between reciprocally connected ensembles [58,59]. By then it had become evident that distant cortical columns often exhibit synchronized firing, sometimes with a near-zero phase lag, in response to peripheral stimulation [60,61]. These results had inspired the temporal binding hypothesis [62]: the notion that neurons contributing to the same representation should fire together or at a constant phase lag (an idea later challenged by others; c.f. Shadlen and Movshon [63]). 

These preliminary ideas gave way to a broad range of neurocomputational accounts that associate temporal coordination with information representation and transfer. One popular hypothesis, promoted by VanRullen and colleagues [7,8,64] and by Moyal and Edelman [39], is that perception is both discrete and cyclical: based on visual phenomena such as the attentional blink [65], they posit that rhythmic fluctuations in electric potential may, during active perception, reflect a periodic sampling process biased by behavioral priorities [39]. In the visual modality, for instance, it has been proposed that alpha band (8–12Hz) oscillations segment information packets encoded in gamma band (>30 Hz) oscillations [49]. The interplay of low and high frequency oscillations has been implicated in gating interareal communication throughout the thalamocortical network [66,67], coordinating spatial and object-based attention [68], and encoding specific information—such as stimulus features [69] and the animal’s position in space [70].

Alongside these neurophysiological perspectives, mathematical theories that equate neuronal representations with structured fluctuations in an underlying state space [11,12,13,17,51,71,72,73,74,75] have emerged. Common to most of these frameworks is their attempt to uncover symbolic structure in continuous, nonstationary dynamics by offering ways to identify separable activity patterns and ensembles. They also share an important underlying assumption: that neural computation is mediated by reproducible [16] spatiotemporal patterns that are both stable enough to be causally effective [76] and varied enough to support a rich behavioral and cognitive repertoire (i.e., they are complex [77,78]). The specifics, however, differ between accounts; depending on context, states have been defined, for instance, as fixed points [18], attractor ruins (defined and discussed in [12,17,52,71]), or saddle points in heteroclinic channels [13]. 

### 2.1. Metastability in the Brain

The coordination dynamics framework [17,46,51,79,80], rooted in earlier work by Haken, Kelso, and Bunz [81], aims to describe and explain how coordinated collective dynamics emerge in complex systems—from neural circuits to interacting agents—on multiple spatiotemporal scales. Key to this approach is the observation that increased integration is marked by lower variance in the phase lag between interacting oscillators [17]. In coordination dynamics, systems that visit attractors are said to be multistable; perturbations are often necessary for such systems to switch between those. The wakeful brain, on the other hand, is thought to be metastable [17]. In this regime, the system dwells in close proximity to a saddle-node bifurcation [82], where one would observe periods of phase-locking interspersed by phase drift. Such dynamics, if stationary in a long enough time interval (relative to the oscillation frequencies in the band of interest), would trace a trajectory that is more space-filling in areas where the phases are independent and less so where they align. In other words, integrative tendencies in the metastable regime remove degrees of freedom, forcing the dynamics onto a lower-dimensional manifold, while segregative tendencies do the opposite [83]. When the two are sufficiently balanced, their interplay may give rise to complex patterns [14,19,84].

Closely related to metastability is the concept of chimera states, observed by Kuramoto and Battogtokh [85] in systems of coupled limit cycle oscillators [86]. In such states, some coalitions are phase locked while others are incoherent [86]. Shanahan [31] observed that such dynamics can arise in networks with “community structure”—that is, ones that contain separable clusters of densely interconnected nodes that make external connections via a relatively small number of hubs [87]. This structure resembles that of the human cortical connectome [88]. Shanahan [31] further showed that the repertoire of metastable states, as measured by coalition entropy [89], could be increased by fine-tuning network modularity; the corresponding increase in representational capacity is one of the hallmarks of criticality (see Section 2.2).

One mathematically explicit account that is compatible with the metastable regime envisioned by coordination dynamics is the winnerless competition framework of Rabinovich and colleagues [11,13]. Spatiotemporal coding in the brain, on this view, hinges on competitive interactions among subnetworks [11]. The phase space trajectory visits sequences of saddle points connected by unstable separatrices; together, they form heteroclinic channels wherein the unstable manifold of one saddle is the stable manifold of the next [90,91]. Such dynamics, in idealized form, are exemplified by the generalized Lotka–Volterra equations [16]; they differ from winner-take-all dynamics, which are characterized by multistability [90]. The winnerless competition model was found to approximate the dynamics observed in olfactory circuits (e.g., in studies showing that repeated exposure to odorants gives rise to reproducible spike sequences that are resistant to perturbations [11]).

A slightly different framework—chaotic itinerancy—postulates that brains chaotically transition between the sites of attractors that were annihilated in a bifurcation [12,52,92,93,94]. The dynamics in the vicinity of such attractive states is described by Tsuda [52] as “a local region of weakly convergent flows” (p. 67). These ordered zones exhibit periodic behavior, which is said to correspond to representational content; the chaotic transitions are thought to reflect searches [52,94]. Chaotic itinerancy is compatible with neurophysiological evidence; commonly cited are the studies of Skarda and Freeman [95,96] showing that unknown odors elicit chaotic transitions between learned configurations [52,94]. Conceptually, chaotic itinerancy is similar to winnerless competition in that both provide a mapping from continuous dynamics to a quasi-discrete, symbolic description (sequences of stable, ordered transients in some subnetworks). The underlying mechanics, however, differ. For one, in winnerless competition, transitions are not always chaotic [94]. In chaotic itinerancy, the saddle points are replaced with convergent flows at the site of an attractor ruin, and the unstable separatrices are replaced with chaotic wandering on some manifold. Importantly, both accounts are compatible with biased competition models of attention [97,98,99,100].

Despite their rigor and plausibility, coordination dynamics, winnerless competition, and chaotic itinerancy are not frequently used to explain or predict the time evolution of oscillatory patterns in real or simulated neural data. In a recent attempt to bridge this gap, Roberts and colleagues [34] looked for metastable structure in the wave patterns produced by a large-scale neural mean-field model [101] based on the human connectome. To study its dynamics, they examined the vector field defined by the phase velocities and observed repetitive wave patterns punctuated by rapid transitions, consistent with a metastable regime. Interestingly, they also found that the transitions were accompanied by desynchronization at lower frequencies, which in behavioral studies often occurs near event boundaries and at stimulus onset [102,103,104].

Repetitive wave patterns such as those observed in neural field models [34,105,106] may subserve a wide variety of cognitive and perceptual processes in the human thalamocortical complex [107]. One classic example is binocular rivalry [108], a visual phenomenon wherein the presentation of competing signals to different eyes results in multistable perceptual shifts (either between the two original signals or some combinations thereof, depending on their content [109]). Lee, Blake, and Heeger [110], for instance, observed traveling waves in BOLD fMRI signals from V1 of human participants as they reported perceptual switching between two oriented gratings; they found that their timing and locations corresponded to those of the perceptual waves during transitions. Electrophysiological studies further suggest that traveling waves initiate perceptual switching [111,112], that changes in natural scenes alter the profile of gamma oscillation phases as well as the direction of their propagation [113], and that moving stimuli evoke direction-specific traveling wave patterns in local field potentials (LFPs) recorded from motion processing areas [114].

Evidence tying large-scale synchronization to the active maintenance of conscious sensory representations is also available. Doesburg, Green, McDonald, and Ward [115], for instance, presented human participants with rivaling natural images and found increased gamma band synchronization—time-locked to background theta oscillations—immediately prior to perceptual switching. This is consistent with the idea that coherent nested oscillations carry representational content (an idea developed mainly by Lisman, Jensen, and their colleagues [116,117]; for a detailed discussion, see Section 3.1). Similarly, Nakatani and van Leeuwen [118] found that transient synchronization between frontal and parietal areas preceded switching. Another recent study, utilizing continuous flash suppression (a technique derived from binocular rivalry, involving the suppression of a static stimulus presented to one eye by an animation presented to the other [119]), found that resets in theta oscillation phase occurred concomitantly with detection [120]; the number of channels phase-locked to posterior activity immediately prior to detection was higher in sites contralateral to the stimulus [120].

Stimulus-related dynamical switching between stable states has been observed in electrophysiological data from different species and modalities, in both conscious and unconscious animals [121,122]. Jones, Fontanini, and Sadacca [123] demonstrated that, in the gustatory cortex of awake rats, natural stimuli reliably evoke sequences of stable firing patterns. The states, which they estimated using a Hidden Markov Model, were separated by brief transitions which were an order of magnitude shorter. Mazor and Laurent [124], similarly, describe the spike rate dynamics in the locust antennal lobe following a long exposure to an odorant as a stimulus-specific transition followed by an “odor-specific fixed point” and a reduction in population synchronization. These stable patterns, just like those observed by Jones and colleagues [123], persisted for periods one order of magnitude longer than the 1–2 second transitions leading to them; stimulus offset resulted in another transient followed by a return to baseline. Interestingly, they also found that the maximal separation between odor-specific patterns occurred at the transitions, rather than following stabilization [124]. 

The evidence surveyed here suggests that representationally meaningful states may manifest as distributed, reproducible oscillatory patterns interspersed by rapid transitions (desynchronization). On some accounts, like coordination dynamics and winnerless competition, those may join up sequentially and hierarchically to form spatiotemporal structures that, when sufficiently strained (e.g., due to nonlinear dependence on other components or to external perturbations [17,125]), dissolve and give way to new configurations spanning different portions of the network [33,35]. It is possible that coherent sensory representations rise to awareness by undergoing causal and temporal integration with a metastable thalamocortical “dynamic core” [59,126], compatibly with both GNW [50] and IIT [42] (this possibility is explored in Section 4).

### 2.2. Useful Computational Properties of Near-Critical Systems

Under what circumstances do biologically plausible neural networks acquire and utilize a large repertoire of complex metastable states? How do they dynamically switch between periods of high and low computational capacity (e.g., wakefulness and sleep)?

Experiments in cellular automata and neural models [23,127] suggest that, on the cusp of a second-order phase transition (a change in an order parameter as a function of some control parameter [128]), systems of interdependent components strike a near-perfect balance between order and disorder [22]. This regime has been referred to as the “edge of chaos” or the “critical point”. The increase in complexity near the critical point can be observed in many physical systems [25]. In neural networks, the balance of excitatory and inhibitory synaptic inputs (E–I ratio), which stays relatively constant on average even when activity varies, can act as a control parameter [129,130]. The notion that brains self-organize to operate near a phase transition—dubbed the criticality hypothesis by Beggs [22]—has recently begun to enjoy increasing popularity, seeing as near-critical networks exhibit characteristics that are conducive to computation. 

One useful computational property of near-critical neural systems is their tendency to propagate perturbations over longer distances compared to subcritical networks. In slices of cortex, the sizes of spontaneous activity bursts (“avalanches”) often exhibit power-law distributions [26]; similar patterns have been observed in spike, LFP, and encephalography data in humans and nonhuman animals [22,25,26]. Poil, van Ooyen, and Linkenkaer-Hansen [131], for instance, found that avalanche length distributions in simulated critical branching processes resembled those computed from bursts of resting-state MEG alpha oscillations (defined based on relative amplitude). Importantly, they also found that temporal correlations persisted on time scales longer than the duration of a single burst [131]. Similarly, Mazzoni and colleagues [132] compared spontaneous activity in leech ganglia and slices of rat hippocampal neurons; both systems, in spite of their differences, exhibited a 1/f power spectrum and long-range temporal correlations. They found that manipulating the E–I ratio by blocking inhibitory pathways increased the temporal correlations. Shew and colleagues [129], likewise, manipulated the E–I ratio in anesthetized rat and awake macaque cortex while recording LFPs; at intermediate ratios, both transmission efficacy (quantified in terms of mutual information) and entropy were maximized. Curiously, similar patterns have also been observed in human BOLD fMRI: the durations of phase-locking periods, for instance, have been reported to follow a power law [28]. While providing mostly circumstantial support for the criticality hypothesis, these findings are fully compatible with a spatiotemporal code based on metastable network states.

Another important characteristic of nonlinear systems poised at the edge of chaos is their improved representational capacity and enhanced ability to generate separable (classifiable) patterns in response to complex input. Haldeman and Beggs [27], for instance, tuned the branching parameter of networks consisting of binary units to the critical point—the value for which the overall number of active units remains relatively constant over time and perturbations do not die down quickly (subcritical) or cause runaway excitation (supercritical). They found that the number of distinct metastable states—defined in this context as output configurations more similar to each other than chance would have it—was maximized at slightly supercritical values, approaching criticality as the network size increased. Kinouchi and Copelli [133] similarly found that, in addition to representational capacity, input sensitivity and dynamic range were maximized near the critical point. Bertschinger and Natschläger [24] also observed increased network-mediated separation (a measure of input-related differentiation in the trajectories that takes into account the expected divergence due to chaotic sensitivity); when they supplied their randomly connected recurrent neural networks with real-time input, they found that, near criticality, the information encoded by their state trajectories was easier to classify. 

To summarize, tuning the balance of excitation and inhibition in recurrent neural networks to near-critical values endows them with complex dynamics and enables (but, importantly, is not sufficient for) meaningful computation. The extension of the range of bidirectional causal interactions, combined with the joint optimization of the system’s sensitivity to perturbation and its tendency to follow stable and precise (i.e., approximately periodic in some subspace and interval) trajectories, increases the number of separable (informative) states that the system may visit.

### 2.3. Quantifying Representational Capacity and Content

The theoretical constructs outlined above allow for estimating representational content and representational capacity in reasonable time for multivariate neural time series data [17,44,45,78,134]. An organizational framework recently introduced by the authors—Dynamical Emergence Theory [78]—sets the stage for the development of empirical measures defined in terms of the geometry or topology [44,45,135] of spatiotemporal patterns in an underlying state space or physical field [136], complementing IIT’s focus on discrete systems and momentary state vectors [42] (see also Section 4.2). This approach, combined with the tools and definitions developed as part of the metastable brain framework, offers a natural discretization algorithm and requires weaker assumptions compared to IIT [137]. It is only assumed (compatibly with the criticality hypothesis [22]) that the wakeful brain may approximate low-order Markovian dynamics, allowing—at least in principle—for the identification of hierarchical metastable states and transitions thereof.

In Geometric Theory [44,45], the conceptual predecessor of our framework [76], state-space trajectories serve as representational primitives [44]. A system’s computational efficacy is a function of the topological complexity of its activity space [45] (that is, the space of all viable trajectories of finite duration that the system may produce [76]). Fekete [45] makes two fundamental assumptions—that the activity space is metric and that it can be parcellated into “semantically- invariant” neighborhoods (equivalence classes) corresponding to distinct representations. The latter notion accords with basic psychophysical data suggesting that percepts are fundamentally discrete (such as the existence of just noticeable differences in every modality [138]), but does not disallow fast, quasi-continuous sequences of stable spatiotemporal patterns (such as those that may underlie auditory perception). The richness of the representational content—that is, the number and grain of the equivalence classes, each corresponding to some quasi-stable representation (e.g., a particular composite percept such as a visual scene)—should fluctuate with the parameters that dictate the topological complexity of its activity space (in the case of neural systems, one such control parameter could be *gain*; see Section 3.3.1). In other words, as Fekete and Edelman [44] put it, the representational capacity of a system corresponds to “the degree to which its activity separates itself into clusters” (p. 811)—or to the number of “holes” in the trajectory space. 

Importantly, the representational structure in complex dynamical systems spans multiple spatial and temporal scales; the measured topological complexity of the activity space thus varies depending on resolution. In the visual system, for instance, percepts normally take tens to hundreds of milliseconds to stabilize and rise to awareness [139] (and sometimes more in edge cases such as binocular rivalry [108]); in many cases, the full complexity of composite representations may only be reflected in trajectories that span seconds of activity. Fekete and Edelman [44] proposed to capture the hierarchical structure of state-space trajectories using persistent homology, which measures the number of separable clusters as a function of scale. To map this complexity to the system’s representational capacity (operationalized experimentally as its level of arousal), Fekete [45] proposed to empirically define a scalar state indicator function (SIF) over the activity space (e.g., in terms of the statistical or geometric complexity of recorded activity [140]). As a proof-of-concept, Fekete, Pitowski, Grinvald, and Omer [140] applied these definitions to voltage-sensitive dye imaging (VSDI) data from monkey V1 during anesthesia, drowsiness, and active perception. They successfully classified the outputs of the SIF for different recorded states, then computed the persistent homology in terms of Betti graphs (constructed from the numbers of k-dimensional holes in the space as a function of scale). They found that, across scales, complexity increased with the level of arousal.

More recently, Tajima and Kanai proposed a similar topological definition of representational capacity [141]. Integrated Information [42], on their account, corresponds to the dimensionality of manifolds reconstructed from empirical observations using delay embedding [141]. Takens’ embedding theorem [142,143], on which this method is based, reflects an important property of nonlinear dynamical systems: it guarantees that if the system satisfies certain conditions and has an attractor, sampling the values at a particular location (e.g., the potential of a participating unit in a neural network) and constructing a trajectory from time-lagged copies of the sample would give a manifold that is topologically equivalent to that of the full system in the original space (in the present example, other neurons that causally interact with the one sampled). It is important to note, however, that the reconstruction of manifolds using delay embedding may not be practical for high-dimensional systems; furthermore, a proper embedding dimension must be selected.

A closely related method for quantifying directional nonlinear causal interactions in complex systems is convergent cross-mapping, developed by Sugihara and colleagues [144,145]. This method, which is related to measures of generalized synchronization [146,147,148], hinges on the observation that if two variables (observations) are causally related, points that are nearby on one reconstructed manifold should also be close on the other; specifically, if the states of a delay-embedded time series can be estimated from another, then the former is causally influenced by the latter [144]. Curiously, it has been shown [149]—in both EEG and fMRI data sets—that using convergent cross-mapping to reconstruct resting state networks gives similar results to using the phase-locking value (PLV) [150] where there exist bidirectional influences. Thus, at least for high sampling rate neural data (e.g., resting state EEG recordings), convergent cross-mapping could be a potent tool for defining the boundaries of functionally interacting assemblies and for tracking the consequences of incoming perturbations. It may also help operationally define the Global Neuronal Workspace as it evolves in time (see Section 4).

These measures can help gauge a system’s representational capacity and demarcate the boundaries between causally interacting subsystems therein; they do not, however, explicitly model the geometry of separable states. This can be easily remedied: empirically, representational capacity and content can both be estimated from multivariate neural data by attempting to infer the metastable state repertoire from phase relationships. In the spirit of coordination dynamics [17], one could compute the relative phase of pairs of time series in a frequency band of interest or, following Roberts and colleagues [34], one could instead compute phase velocity vectors. Specifically, low variance in the former measure would signify that the joint trajectory is highly precise (i.e., less space-filling—lower dimensional), indicating a dwelling at an attractive state which could potentially (but not necessarily) carry information. The information content of the estimated state sequences should be proportional to their complexity and can be assessed using standard classification methods. Performed over multiple narrow frequency bands, this procedure may also allow for the detection of hierarchical spatiotemporal structure (e.g., high frequency packet sequences nested in lower frequency ones [139]; see Section 3.1). Such nested sequences would be assigned a higher representational capacity score by the persistent homology procedure of Fekete [45] when they have a nested structure, compared to both unstructured and trivially structured trajectories. Although empirical procedures such as these cannot perfectly extract the underlying dynamics of noisy electrophysiological data, they could help narrow down the search space and generate more informative features for classification.

To recap, the repertoire of metastable attractive states can be estimated from neural time series data by, e.g., computing pairwise phase lags [17] for different intervals and frequency bands. Representational capacity can be estimated using persistent homology [44,45] or by directly computing the dimensionality of the measured or delay embedded manifold [141]. Representational content can be characterized by defining similarity metrics over the nested metastable oscillatory states (based, e.g., on their state-space geometry or the spatial distribution of the participating components) and by classifying them where possible [78]. The boundaries between systems and states can be estimated—for a particular finite time interval—by computing the strength and the directionality of nonlinear causal interactions, as measured by convergent cross-mapping [144] or by similar theory-driven techniques (e.g., information geometry [151,152,153]).

## 3. Visual Representations as Metastable Oscillatory States

It has long been understood that bidirectional thalamocortical interactions (often referred to as recurrent or reentrant [19,58,154]) underlie complex cognitive operations that necessitate sustained, distributed processing [5,155]. In the primate visual system, the pulvinar—a higher-order thalamic nucleus [156,157]—mediates sensorimotor processes as diverse as saccade initiation [158,159], grasping and reaching [160], feature binding [161], and visual detection [162]. Several recent results—showing, for instance, that the pulvinar helps propagate stimulus-evoked activity and synchronizes cortical local field oscillations (LFOs) [163,164,165]—have led to a resurgence of interest in its possible contributions to cortical information processing. These recent developments make the primate visual system an ideal case study: its oscillatory dynamics are now within our purview, its circuitry is relatively well-known [156,157,166,167,168], and there exists a large body of research relating these to conscious perception and behavior (discussed in Section 3.3.2). 

The primary aim of this section is to relate the metastable brain framework to the thalamocortical dynamics underlying visual perception. We begin by reviewing a set of interrelated hypotheses implicating hierarchical spatiotemporal coordination in mediating neural information transfer and maintenance. Specifically, we briefly outline the communication through coherence [36,37] and (pulsed) gating by inhibition [47,48,169] accounts and describe recent advances, with an emphasis on the communication through nested oscillations hypothesis [49]. Since these proposals were discussed elsewhere [139,170], we focus on evaluating their plausibility based on visual thalamocortical circuit motifs and pertinent electrophysiological results.

### 3.1. Nested Oscillations: A Spatiotemporal Neural Code?

In recent decades, it has become evident that neural oscillations support information representation, transfer, storage, and retrieval [170,171,172]. For one, local field oscillations can regulate spike timing precision [173]: the synchronization of subthreshold input currents [174] may result in a periodic shift in the probability of firing, which typically enhances the quality of long-distance transmission (as precise firing in a sending population tends to enhance both firing rates and spike reliability in a target population [172,175,176]). Repetitive firing at a specific phase could also be utilized for gain modulation (perhaps in the service of attentional processes [177]); increasing the net excitation in an ensemble and bringing it closer to criticality may facilitate the formation of more complex metastable patterns (Hebbian assemblies [54]) spanning larger areas.

Ample electrophysiological work suggests that the timing of spikes relative to slow (under 12Hz) background LFOs encodes information. In the visual cortex, for instance, it was demonstrated that spike timing can help differentiate between input features that evoke similar firing rates (“spike-phase coding” [69,178,179,180]). Another classic example comes from the hippocampal system: as a rat traverses a location within the receptive field of a particular place cell, it begins to fire at earlier theta phases (the “theta phase precession” [181]). This phenomenon, together with findings suggesting that gamma spike-field coherence does not significantly alter the information content of population responses [116], has led Lisman [116] to propose that spikes occurring within the same gamma cycle encode individual spatial positions or events; consecutive gamma cycles nested within a single theta cycle would thus encode sequences of events. Such a mechanism, he argued, could serve to sequentially organize discrete units of information (for a more recent formulation, see Lisman and Jensen [117]). 

Similar ideas have been extended to the visual system in light of evidence linking low-frequency rhythms to the deployment of spatial attention [182] and to visual detection performance [48,183]. In a landmark EEG study, Mathewson and colleagues [183] found that when the onset of a target stimulus coincided with the negative peak of alpha oscillations at posterior sites, participants were less likely to detect it. These results have led them to propose a pulsed inhibition account of the alpha rhythm: when alpha power is high, detection is optimized when stimuli appear at its excitatory phase; when alpha power is low, input is likely to be processed regardless of its timing with respect to the oscillation [48]. Their account is compatible with the gating by inhibition hypothesis of Jensen and Mazaheri [47], which postulates that alpha LFOs reflect a periodic inhibition of neuronal populations and confine gamma cycles to their troughs. Subsequent studies have indeed found a coupling between alpha phase and gamma amplitude [169] as well as an inverse relationship between alpha and gamma amplitudes (e.g., in awake macaques [184]).

The coupling between spike timing and slow LFO phase may also extend to the gamma band—an idea that has received a thorough treatment in Fries’ communication through coherence framework [36,37]. On this account, the gamma rhythm reflects brief periods of relative excitation followed by periods of inhibition; spiking is limited to short windows of opportunity within each gamma cycle [37]. Since spikes are more impactful when postsynaptic targets are gamma-coherent with the sending population, effective connectivity and selective routing are thought to be achieved through interareal gamma synchronization (which needs not be zero-phase) [37]. Specifically, LFO phase differences between coupled populations are assumed to align such that inputs from a sending ensemble arrive precisely when the receiving ensemble is at its most excitable phase [37]. 

As Voloh and Womelsdorf [185] point out, this coordination may be achieved by phase resetting—that is, through a rapid realignment of the phases of interacting components or sites. This indicator of dynamic network reconfiguration, they note, has been observed in various species and contexts (e.g., in primates performing a visual attention task [186]). Similarly, in EEG studies, stimulus onsets and event boundaries [103] are often accompanied by desynchronization and phase resetting in various frequency bands [102]. These transitions, on some accounts, may contribute to the formation of event-related potentials [187]. Coordination between two neurons or populations may also be achieved through weak coupling, i.e., by the repeated adjustment of their instantaneous frequencies to achieve an approximate locking; this behavior was observed, for instance, in the case of gamma oscillations in V1 microcolumns [188]. This push–pull relationship between the phases may be used to estimate the metastable state repertoire from neural time series data.

In recent years, gating by inhibition and communication through coherence have morphed into a more comprehensive hypothesis, tackling the very nature of the neural code. Bonnefond, Kastner, and Jensen [49], in their communication through nested oscillations account, describe a coding scheme wherein slow phase synchronization augments gamma spike-field coherence and routes (or blocks) information to specific destinations. In keeping with both of its predecessors, this account assumes that gamma packets carry information while slower oscillations strictly route it. Luczak, McNaughton, and Harris [139] proposed a similar coding scheme that is, in some respects, also analogous to winnerless competition: in their view, sequentially organized stimulus-evoked spike patterns constitute stackable representational units. Importantly, they note that continuous stimulation often evokes transient bursts that persist throughout the presentation [189] and that representational content carried in the population response is elaborated over time, with more complex features becoming decodable later in the cycle [190,191]. This elaboration, which should be reflected in increased topological complexity (due to the diversification of spike timing following onset), is likely to be enhanced by increases in gain [192]. Luczak and colleagues [139] also point out that stimulus-evoked and spontaneous packets are detected first in the granular and deep layers, respectively, suggesting that the former propagate away from the periphery whereas the latter travel towards it (see Section 3.2.2).

We note that there exists a clear analogy between these neurophysiological descriptions and the constructs of the metastable brain framework. As seen in previous sections, following a stimulus-evoked perturbation, activity in some visual cortical populations will relax to a phase-synchronized state in some bands (or to a sequence thereof, perhaps involving different subpopulations [31]). In geometric terms, each such transient stabilization amounts to a confinement of a segment of the state-space trajectory to an attractive zone. Since those are determined solely by the nonlinear dependencies among the constituent parts, they parcellate the activity space into intrinsic (observer-independent [193]) equivalence classes that evolve over time (e.g., due to synaptic learning [194]). Sets of metastable states and transitions (e.g., in winnerless competition, saddle points, and separatrices) can be stacked hierarchically by nesting shorter sequences within longer ones. The precision of the coordinated LFP and spiking activity during stable periods (which corresponds to the strength of the coupling and can be estimated from instantaneous phase relationships) should allow for the faithful reproduction of these spatiotemporal patterns when a representation is actively maintained.

### 3.2. The Visual Alpha-Gamma Code

To evaluate the plausibility of this unified perspective and integrate it with electrophysiological data, we must first outline the circuitry of the visual thalamocortical system. We briefly describe the major pathways within and between visual cortical microcolumns, the pulvinar, and the thalamic reticular nucleus (TRN), skipping the lateral geniculate nucleus (LGN) for brevity [195,196,197,198]. We then explore the contributions of different thalamic nuclei and cortical interneuron classes to the generation of nested alpha and gamma rhythms.

#### 3.2.1. On the Organization of the Visual Thalamocortical System

Like geniculostriate projections, intercolumnar V1 connections that carry information away from the periphery tend to terminate in layer 4 of their target microcolumns [167]. From there, the classic feedforward pathway continues internally to layer 3 pyramidal cells, which proceed to excite layer 4 pyramidal cells in higher areas [166]. Interareal feedback connections tend to originate in layer 6 pyramidal cells and target layer 1 interneurons and dendrites, with occasional terminations in supragranular and deep layers (as exemplified, e.g., by V4 to V2 projections in the macaque [199]) [167]. The infragranular layers contain separate populations of corticothalamic and cortical feedback neurons; in many mammalian species, layer 5 thalamocortical cells target higher-order thalamus, whereas layer 6 pyramidal cells target the thalamic reticular nucleus (TRN) and the LGN [197]. 

Within the microcolumn, supragranular pyramidal cells and interneurons project strongly to layer 5 and weakly to layer 6; the interneurons are more integrated with feedback pathways, whereas the pyramidal cells also tend to have extensive intracolumnar connections [167,197,200]. One motif that persists in different species is the presence of intralaminar inhibition: fast spiking interneurons are often reciprocally connected to principal cells in the same layer [201,202]. Those may give rise to gamma oscillations and to their synchronization by implementing a pyramidal-interneuron gamma (PING) mechanism, whereby gamma oscillations arise from the rapid alternation of excitation and inhibition [203].

The pulvinar, being a higher-order thalamic nucleus, receives mostly cortical input [168]. In humans, the ventral pulvinar connects to occipital and temporal cortex, whereas the dorsal pulvinar connects to frontal and parietal areas [204]. Low-level visual cortex targets the inferolateral pulvinar, parietal inputs have more lateromedial targets, and frontal, cingulate, and limbic areas connect mostly to its medial subdivision [205,206]. Projections from the ventral stream to the pulvinar advance such that receptive fields (RFs) become more complex in more medial portions [156,207,208]; also, if two cortical areas are interconnected, they likely project to nearby pulvinar targets [156]. Layer 5 pyramidal cells that project to layers 2–4B in V1 do not project subcortically, while those that connect mostly within layers 5 and 6 often do [203,209]. Similarly, layer 6 cortico-pulvinar and cortico-cortical cells form distinct populations [166]. 

Driving and modulatory cortico-pulvinar inputs likely originate in layer 5 and 6 pyramidal cells, respectively [210]. Their pulvino-cortical counterparts tend to terminate in layers 4 and 1 in visual cortex in a similar fashion [211]. Projections from layer 5 of V1 to the pulvinar have thus been thought of as indirect feedforward connections from deep pyramidal cells in one level to supragranular cells in the next, with layer 6 projections forming alternative feedback connections that, like their cortico-cortical counterparts, terminate in the lower level’s superficial layers [156,157,168]. This division may also be reflected in the core–matrix organization principle: calbindin-immunoreactive (matrix) thalamocortical cells project to the superficial layers and receive spatially diffuse input, whereas parvalbumin-immunoreactive (core) cells target the middle layers and receive more specific input [212]. Corticopulvinar projections, however, are not likely to relay topographical information [207]; rather, they may subserve spatial attention and other high-level modulatory processes [213].

Intrathalamic inhibition is largely supplied by the reticular nucleus (TRN), which covers the dorsal thalamus and consists of sectors associated with different sensory modalities [214]. Each of these connects to both first- and higher-order thalamic nuclei and regulates their firing modes (burst and tonic firing [215]) by providing or withholding inhibition in response to signals originating in various cortical and subcortical areas [157,216]. TRN-mediated switching between the burst and tonic firing modes may serve diverse functional roles—from blocking the relay of cortical input through the thalamus to coordinating oscillations in targeted cortical microcolumns. 

The visual subdivisions of the TRN contain topographically organized representations of the visual field [168]. Crick [217], therefore, hypothesized that TRN cells exert precise control on their first-order targets, amplifying or attenuating responses in accordance with attentional priorities (this view has been refined in recent years [218,219,220]). As Saalmann and Kastner [157] point out, the TRN receives cholinergic inputs from brainstem structures as well as collaterals from first-order nuclei and thalamocortical feedback axons, suggesting that the inhibition it provides could be either specific (e.g., to precisely modulate gain) or nonspecific (e.g., to facilitate broad changes in responsivity or alertness). Geniculocortical and corticogeniculate cells both send collaterals to TRN [221], as do feedback cortico-pulvinar connections from layer 6 [222]. Feedforward pulvino-cortical axons, as a general rule, also branch and send collaterals to cells in the inner visual TRN [208] (p. 145); those, in turn, send modulatory projections back to the pulvinar [157].

#### 3.2.2. Thalamic and Cortical Contributions to Alpha-Gamma Dynamics

Throughout the years, various thalamic nuclei have been implicated in the modulation of low-frequency cortical oscillations [223]; in fact, as Bollimunta, Mo, Schroeder, and Ding [224] point out, the idea that they can be generated in the cortical microcolumn without thalamic influence is relatively new. Lorincz and colleagues [225], for instance, found that the peak spike rates of some high-threshold bursting LGN neurons coincided with the negative peak of the alpha band LFOs in the LGN and PGN (visual TRN) of awake cats. Combined with earlier results indicating that corticogeniculate feedback synchronizes the firing of LGN relay cells with matching RFs in the alpha band [226], these findings suggest that modulatory corticogeniculate inputs coordinate the activity of LGN relay cells involved in representing specific visual features (i.e., increase the precision of their spikes) so as to enhance the rhythmic drive to cortex and thus induce stronger gamma oscillations [227] in populations of coupled fast spiking interneurons and pyramidal cells within it.

Gamma oscillations are not as common in first-order thalamic nuclei as they are in cortex [228]. Bastos and colleagues [228] recorded from awake macaque LGN and V1 cells with overlapping RFs and showed that, unlike in V1, visual stimulation does not induce gamma oscillations in the LGN. Alpha- and beta-band synchronization, however, was found, the former led by corticogeniculate feedback and the latter by geniculocortical drive, lending further credence to the notion that alpha- or beta-synchronized LGN spikes drive cortex more efficiently and contribute to the genesis of cortical gamma rhythms [228]. The pulvinar has also been shown to modulate gamma oscillations in cortex. In one study [229], inactivating the cat lateral posterior–pulvinar complex either significantly reduced or increased spike rates and gamma power in visual cortex, depending on whether the cortical RFs did or did not overlap with those of the inactivated pulvinar cells, respectively. This pattern foreshadows later results in primates [164] and complements earlier studies wherein both multiunit and LFP gamma power increased following the presentation of oriented bars and gratings, mostly in supragranular layers [230,231]. Plausible models of the visual thalamocortical system also suggest that spike timing precision in thalamocortical or feedforward cortical cells plays a role in establishing long-range phase alignment [232,233,234]; some were shown to exhibit high-frequency synchronization as the excitability of thalamocortical relays surpassed a certain threshold [232,233]. Taken together, these results suggest that coherent gamma activity, triggered by rhythmic feedforward drive, contributes to the maintenance of stimulus-evoked representations throughout the visual system.

Alpha oscillations, according to Jensen and Mazaheri [47], may be generated within the visual cortical microcolumn by somatostatin-expressing (SOM) interneurons in the supragranular layers. This hypothesis fits comfortably with the existence of reciprocal connections between those regular spiking and low-threshold spiking interneurons [202] and feedback-recipient (via their superficial dendrites [196]) layer 5 pyramidal cells. These superficial SOM interneurons, which tend to resonate at lower frequencies [227], may periodically inhibit their layer 5 targets. The laminar profile of band-specific spike-field coherence seems compatible with this scenario: in the alpha band, it was observed to be higher in the deep layers, whereas in the gamma band, it dominated in superficial layers [235]. 

The coupling sometimes observed between alpha phase and gamma power [169] may arise from the unique connectivity patterns of different cortical interneuron populations. SOM interneurons in the supragranular layers of mouse visual cortex, for instance, avoid inhibiting each other and target other interneuron populations instead; parvalbumin-expressing (PV) interneurons, which include fast spiking basket cells, tend to target each other [236]. Another class of interneurons (VIP), more common in layers 2–4 than in layer 5, preferentially targets SOM interneurons [236]. It is therefore plausible that alpha-rhythmic (dis)inhibition could be imposed on supragranular pyramidal cells either directly, by the projections of SOM interneurons to their dendrites, or indirectly, by SOM-to-PV connections. In either case, gamma oscillations generated by coupled PV interneurons and pyramidal cells would be coupled to SOM cell activity, which may in turn be dampened by VIP interneurons (which are the type primarily targeted by geniculostriate inputs [197]). Such dynamics may give rise to nested alpha and gamma LFOs [49], regulated by both cortical and thalamic signals.

It is relatively well-established that, in awake primates, alpha oscillations tend to propagate towards the periphery (in the “feedback” direction) while gamma oscillations propagate away from it (“feedforward”) [237,238,239]. In a landmark study, Van Kerkoerle and colleagues [240] recorded from macaque V1 and V4 during a figure-ground segmentation task. They found that gamma (but not alpha) LFOs were stronger when the figure was in the RF, indicating that they may be involved in the active representation of salient visual inputs. LFP alpha power was strongest in layers 1, 2, 5, and 6, whereas gamma power was most pronounced in layer 3. More importantly, they showed that alpha oscillations were driven by layers 1, 2, and 5, whereas gamma activity propagated from layer 4 to superficial and deep layers. This pattern was also confirmed using causality measures [240]. Other studies, however, complicate the interpretation of these results: Bollimunta, Chen, Schroeder, and Ding [241] showed that alpha sources vary between visual areas, with mostly deep sources in V2 and V4 and supragranular sources in IT. Haegens and colleagues [242] showed that alpha generators in V1 were strongest in the supragranular layers whereas LFP alpha power was greatest in the infragranular layers. In a human ECoG study, Halgren and colleagues [243] found that during quiet wakefulness alpha LFOs propagated from anterosuperior to inferoposterior cortical areas; pulvinar alpha rhythms were found to be more synchronized with cortical than with pulvinar high gamma power.

It thus seems plausible that—at least in V1—superficial SOM interneurons, driven by excitatory top-down feedback from layer 6 pyramidal cells in higher columns and from pulvino-cortical backward projections, may drive alpha oscillations in their microcolumn and maintain them in tandem with layer 5 pyramidal cells to which they are reciprocally connected [244]. Cortical gamma oscillations, on the other hand, seem to be primarily generated through the interactions of fast spiking interneurons and pyramidal cells in the middle layers (and enhanced by synchronized feedforward input). By default, alpha oscillations seem to propagate in the feedback direction [243,245]; however, pulvinar-mediated deviations from this regime may play a role in routing gamma packets downstream [246,247].

### 3.3. Attention-Gated Visual Processing through Pulvinocortical Coordination

The findings examined up to this point clarify the mechanisms and typical propagation patterns of alpha and gamma oscillations in the visual system. To understand how their coordination could support the formation and maintenance of metastable visual representations, we now turn to evidence indicating that the pulvinar facilitates cortical phase alignment [156] in the service of diverse cognitive operations. Since many of these results have been reviewed in detail elsewhere [213], we use them here primarily as evidence supporting the metastable brain framework. 

#### 3.3.1. The Pulvinar Coordinates Alpha and Gamma Oscillations in Awake Primates

Recent years have seen a surge in electrophysiological work indicating that the pulvinar mediates shifts in visual attention by synchronizing cortical alpha and gamma oscillations and by enhancing spike-field coherence. In a seminal study, Saalmann and colleagues [163] recorded from macaque pulvinar, V4, and temporo-occipital cortex (TEO) neurons with overlapping RFs. They found that when a spatial predictive cue was in their RF, pulvinar spike rates and alpha spike-field coherence were elevated; at target onset, both increased when the monkey attended to the RF location. Importantly, they also observed that attention to the RF enhanced coherence between V4 and TEO LFPs in the alpha and gamma bands prior to stimulus onset and increased alpha-gamma phase–amplitude coupling between the two areas. The pulvinar’s influence on alpha oscillations in V4 was maximal during the delay period when the cue was in the RF [163]. These results indicate that attention-mediated disinhibition in the pulvinar (which may result in tonic firing [215]) enhances the precision of cortical spikes across visual cortical areas through alpha- and gamma band oscillatory synchronization (to the effect of maximizing the impact of anticipated behaviorally relevant stimulation), compatibly with the communication through nested oscillations account.

Similarly, Purushothaman and colleagues [164] recorded from galago layer 2–3 V1 and lateral pulvinar cells with overlapping spatial RFs. The cortical neurons responded to sinusoidal gratings in their RF with phasic firing followed by tonic firing (as is typical for regular spiking pyramidal cells). When pulvinar cells were inactivated by a muscimol injection, visual evoked responses and baseline firing rates in the V1 cells were significantly reduced; LGN responses were not affected by the injection [164]. V1 responses to gratings increased twofold in neurons whose RFs completely overlapped with the RFs of the excited lateral pulvinar cells but were suppressed in those whose RFs only partly overlapped (perhaps due to lateral inhibition in cortex) [164]. As they point out, these findings demonstrate that corticogeniculate input to V1 cannot strongly drive supragranular cells when the lateral pulvinar is decommissioned [164].

More recently, Zhou and colleagues [165] recorded from V4, IT, and ventrolateral pulvinar of rhesus monkeys performing a change detection task. They found that Pulvinar cells exhibited object selectivity and their spatial RFs were larger than in V4 but smaller than in IT; spike-field coherence in V4 and IT cells was higher in the gamma band (and lower in the alpha band, but only in V4) when a target was in their shared RF. Like in previous studies, gamma coherence was found between the pulvinar and V4 and between V4 and IT when the attended stimulus was in a shared spatial RF [165]; also, notably, cortical gamma power was positively correlated (and alpha power negatively correlated) with pulvinar firing rates, which may indicate a switch to the tonic firing mode ([248], fig. 4). Pulvinar deactivation with muscimol caused an increase in cortical low frequency (<20 Hz) power both at baseline and during stimulus presentation, and V4 (but not IT) responses to attended targets were weaker than those evoked by unattended distractors prior to the injection. They also found that V4 led the pulvinar in the gamma band and vice versa in the alpha band [165], suggesting that the pulvinar aligns the phases of cortical alpha oscillations (for a more exhaustive interpretation of these results and their underlying mechanisms, see Halassa and Kastner [213]).

How, specifically, might alpha-rhythmic top-down activity induce gamma synchronization in visual cortex? Quax, Jensen, and Tiesinga [246] examined the plausibility of a simple pulvinar-mediated coordination mechanism (similar to that of Jensen, Gips, Bergmann, and Bonnefond [66]) in a model implemented with Izhikevich neurons [249]. They constructed PING modules [203] and injected alpha-rhythmic drive to fast spiking units in unidirectionally connected modules. Changes in the alpha phase differences between the populations modified the gamma coherence between them, as well as spike rates and gamma spike-field coherence in the receiving module. Specifically, they found that communication was optimized when the sending populations led the receiving ones by approximately 90° [246]. They also found that increases in alpha amplitude had one of two effects: when the sending population led the receiving population, they enhanced gamma coherence; in the opposite case, they decreased it. These results suggest that feedforward cortico-pulvino-cortical connections could, in principle, promote interareal gamma synchronization by injecting a receiving column with a phase-delayed version of the alpha-rhythmic layer 5 pyramidal activity in a sending column. With the relevant pulvinar cells inactivated or in burst mode, higher columns would be more likely to lead lower columns in the alpha band and disrupt the forward thrust of coherent gamma activity.

More recently, Jaramillo, Meijas, and Wang [247] reproduced many of these results in a set of spiking models of the macaque pulvinocortical system, all containing two bidirectionally connected cortical modules (each responsive to one of two stimuli) and a pulvinar module. Pulvinocortical connections were similar to those of the canonical microcircuit outlined earlier, with feedback connections targeting a TRN-like inhibitory module. By manipulating pulvinar excitability (gain), they showed that the pulvinar could facilitate or prevent convergence to a persistent attractive state spanning both cortical modules. Although they did not explicitly model switches between the burst and tonic firing modes due to inhibitory input from the TRN [168,215], these results are nonetheless suggestive: a laminar version of the model, with separate cortical alpha and gamma generators, imitated previous findings [165]. Disabling the pulvinar resulted in increased alpha oscillations in units corresponding to deep pyramidal cells (disinhibited due to the absence of pulvinar input to superficial inhibitory interneurons that project to them [247]; in previous sections, we identified those as SOM interneurons [202]). The apparent agreement between the anatomical, functional, and modeling data surveyed here suggests that the pulvinar regulates metastability in distributed thalamocortical ensembles by altering alpha phase and power in its target cortical microcolumns (that, in turn, may modulate gamma power and coherence [49,66,184,239]).

#### 3.3.2. Pulvinocortical Interactions Support Conscious Perception and Behavior

The behavioral consequences of pulvinar damage have been studied extensively in primates. Chalupa, Coyle, and Lindsley [250], for instance, found that monkeys with inferior pulvinar lesions were slower to react in a visual pattern discrimination task and had difficulty learning briefly presented novel patterns; performance was further impaired when distractors were added. Ungerleider and Christensen [158,159] showed that bilateral pulvinar lesions (sparing the anterior portion) caused monkeys to fixate for longer periods of time and to stop scanning areas that did not contain stimuli, suggesting that their ability to shift attention away from salient input was impaired (similar behavioral symptoms were later reported in a pulvinar inactivation study [251]). Similarly, Desimone, Wessinger, Thomas, and Schneider [252] deactivated the posterior lateral pulvinar (an area connected to IT and V4) of macaques performing a cued color discrimination task. When a single stimulus was presented, pulvinar deactivation resulted in only a small reduction in response accuracy; when a target and a distractor were presented simultaneously to opposite hemifields, performance was significantly impaired when the target occupied the contralesional field [252].

Humans with pulvinar lesions often suffer from spatial neglect [253], not unlike that caused by damage to the inferior parietal lobe [254]. Like monkeys, they present with slower responses to stimuli in the affected visual field [255]. Karnath, Himmelbach, and Rorden [256], for instance, examined anatomical brain images of human stroke patients with unilateral right hemispheric lesions and spatial neglect and found that the lesions primarily affected the pulvinar. Arend and colleagues [161] showed that a patient with anterior and ventral pulvinar lesions had difficulty disengaging attention from affected areas of the visual field in a task requiring rapid spatial attention shifts (but had no problem processing or shifting their gaze to them); curiously, when presented with a composite target surrounded by distractors, the patient made feature binding errors (which were absent when stimuli were presented sequentially). A different patient in the same study, presenting with a posterior medial pulvinar lesion, showed a strikingly different pattern: he had difficulties processing objects within his impaired field (even when no spatial shifts were required) and made binding errors when objects were presented sequentially [161]. Letter-color illusory conjunctions following pulvinar lesions have also been observed [257].

Electrophysiological and neuroimaging studies in healthy primates provide additional clues that further implicate the pulvinar in biasing conscious and attentive processing. Petersen, Robinson, and Keys [254], for instance, explored the response properties of cells in the inferior, lateral, and dorsomedial pulvinar of rhesus monkeys; increased activity in the dorsomedial pulvinar, which is reciprocally connected to posterior parietal cortex (an area implicated in orienting spatial attention and in initiating reflexive exploratory saccades [258]) was observed immediately prior to saccades, as well as when peripheral stimuli were covertly attended [259]. In a landmark study, Wilke, Mueller, and Leopold [162] examined the effects of perceptual switching on responses in LGN, pulvinar, and V1 of macaques trained to report stimulus visibility by pulling a lever. They used a variant of the flash suppression technique that, in humans, is known to induce temporary unawareness of salient stimuli for extended periods of time (sometimes on the order of seconds) [260]. They found that the majority of pulvinar cells exhibited reduced firing rates during suppression, similarly to when the stimulus was actually removed; LGN spike rates, on the other hand, were not affected by the suppression. Correlations between pulvinar firing rates and trial-wise changes in detection and confidence were found in other studies [261,262,263] (cf. Storm and colleagues [264]).

While the pulvinar is known mostly for its role in visual processing, its contributions to orienting across modalities [265], auditory and somatosensory processing [266], perceptual and motor decision-making [160], and multimodal integration (due mostly to the diverse connections of its medial division [213,267,268]) are relatively underexplored. In the cat, auditory and somatosensory evoked responses were found in the posterior ventrolateral and anterior ventromedial pulvinar, respectively [268], and visual-somatosensory units were also documented [269]. Yirmiya and Hocherman [266], for instance, recorded from the medial, lateral, and inferior pulvinar of rhesus monkeys trained to manipulate a lever in response to auditory tones. While some pulvinar cells in their study exhibited selectivity to specific tones, they fired only when the monkeys were required to produce a motor response; in other words, mere exposure to the tones was not enough to elicit a response in most cases [266]. Other pulvinar neurons responded during specific stages of arm movements, but only when the monkeys were performing task-relevant actions (and not, e.g., during spontaneous grooming [266]). Similar behavior was observed by Acuña and colleagues [160] in behaving capuchins: medial pulvinar neurons were found to be more active during intentional (as opposed to spontaneous) eye and muscle movements. In a different study on behaving macaques, posterior pulvinar cells were observed to fire hundreds of milliseconds prior to the initiation of reaching movements; they increased their firing during the movement regardless of its direction [270]. 

This evidence dovetails with findings from the more well-studied inferior and lateral divisions, showing impairments in both covert and overt voluntary shifts of attention following pulvinar lesions. Since the cortical connections of the medial pulvinar exhibit a similar laminar organization to that observed in its more visual divisions, they may likewise facilitate coherence and synchronization in their targets. The same functional logic that allows the pulvinar to arbitrate between competing visual representations (e.g., during rivalry or visual search) may also contribute to the deliberation that often precedes the execution of volitional motor responses. 

## 4. A Causally Integrated Thalamocortical Workspace

The findings reviewed in the previous section strongly suggest that the active maintenance of neural representations is subserved by the sequential organization of nested oscillatory patterns spanning different subnetworks. The strong coupling of spikes to the phase of slow and fast oscillations (which are themselves aligned) would render these patterns more precise and reproducible. Although evidence tying this coordination to behavioral measures of visual attention and awareness is plentiful, it is not yet clear how the information encoded in specialized subsets of the thalamocortical network is combined globally to form composite representations (e.g., in the case of audiovisual integration or visuomotor coordination). In this final section, we explore open questions and future research directions that arise from adding another set of theoretical considerations to the mix—those developed in more general theories of cognition and consciousness (IIT 3.0 [42], GNW [50], and the Free Energy Principle [53]).

### 4.1. The Metastable Thalamocortical Core as a Global Neuronal Workspace

The Global Neuronal Workspace framework [50,271,272], rooted in earlier philosophical work by Baars [273], posits that thalamocortical loops and cortical pyramidal cells with long-range axons encode conscious representations in their sustained firing patterns [50]. On this view, such activations are sparse; that is, only a small subnetwork is excited at any particular time interval. In most formulations of the theory other parts of the system are said to be inhibited [50]; Werner [126], however, correctly observed that nonparticipating neurons would exhibit spontaneous firing, compatibly with the account developed in previous sections. Ensembles that produce spontaneous, noninformative activity would contribute no representational content, either due to the absence of stable spatiotemporal structure or due to being independent of (operationally, out of sync with) a temporally integrated “dynamic core” [35,59]). These two criteria may account for the fact that local synchronization only occasionally gives rise to conscious perception [274,275].

GNW is also compatible with the criticality hypothesis [22]. In neural systems nearing a critical phase transition (whose computational properties were described in Section 2.2), external perturbations are more likely to give rise to ignition—a phenomenon described intuitively by Dehaene and others [50,274] as the emergence of sustained, reverberating activity spanning large swathes of the thalamocortical system once stimulation surpasses a threshold. Of particular empirical interest is the concept of intrinsic ignition, defined by Deco and Kringelbach [276] as the capacity of a particular network element to “propagate neuronal activity to other regions in a given brain state.” (p. 962). This new formalism allows for empirically estimating connectivity matrices based on cooccurring discretized events (e.g., spikes), supplementing the methods described earlier. Those connectivity matrices can then be used to identify hierarchical structure in the dynamics [276].

A functional architecture similar to that described by GNW must arise from any formalism purporting to solve the binding problem [38,277,278,279]—that is, to explain how composite percepts are jointly maintained by distributed neural populations. Although GNW does not explicitly specify what criteria render a set of neurons or a subset of the underlying field a “stable and reproducible brain-scale assembly” [272] (p. 80), sound dynamical extensions of GNW exist. One such model, due to Wallace [280], defines network connection strengths in terms of mutual information, compatibly with the topological measures introduced earlier and with the metastable brain framework. On this account, rapid changes in nonlinear dependence underlie the formation of functional networks [126]. Future work could expand on this formalism and integrate it with recent perspectives [33,35].

It is possible, in keeping with these general principles, that large-scale functional networks (such as those observed at rest) reflect a weakly informative, basic attractive state with which additional complex representations may be integrated (e.g., via temporal coordination). For instance, Barttfeld and colleagues [281] demonstrated that resting state networks during anesthesia exhibit lower complexity and have a relatively rigid, smaller state repertoire. The unconscious state, on their account, corresponds to an attractor whose shape is dictated mostly by structural connectivity patterns; the brain, they argue, chaotically explores its immediate vicinity. Others have focused on resting state networks in the wakeful state; Deco, Kringelbach, Jirsa, and Ritter [35], for example, constructed a neural mass model and fitted it to BOLD fMRI time series. They used the model to uncover a set of regions whose oscillations led activity in the rest of the brain—the “dynamic core” [35]. Importantly, they found that the model best fit the resting state data when the former was near a Hopf bifurcation (i.e., near criticality; see Section 2.2), where the repertoire of metastable states is maximized [27]. By combining these techniques with nonlinear causality measures [144,145,282] to track the time evolution of the metastable core, future studies may investigate the behavioral consequences of transient changes in interareal integration. The absence of such integration may preclude conscious detection, in accordance with GNW.

### 4.2. Nonlinear Causal Interactions and Integrated Information

In its third iteration, Integrated Information Theory remains one of the most mathematically explicit theories of consciousness [42]. However, any attempt to extend it to continuous systems exhibiting metastable wave dynamics may pose significant challenges [283]. As Barrett and Mediano [137] point out, the definition of Ф (the “amount” of consciousness, which corresponds to the system’s representational capacity [284,285]) is not axiomatically constrained, and the current definition [42] assumes that momentary state vectors are informative by virtue of constraining past and future states. Although neural state transition probabilities may depend on the system’s recent history (as “brain dynamics are non-Markovian at many levels” [137]), this definition can nonetheless be applied in empirical settings. In fact, many ad-hoc measures circumventing the limitations of the formalism have been proposed [286,287,288].

While the assumption that conscious percepts consist of discrete and composite building blocks is sound (e.g., due to the existence of just noticeable differences [138]), the axioms of IIT do not prescribe one principled way to infer those from continuous neural data (or field models) [137,289]. In effect, IIT’s current formalism sidesteps the discretization problem inherent to mapping brain dynamics to separable, hierarchical representational states. One promising extension, recently proposed by Esteban and colleagues [290], maps the constructs of IIT to their possible manifestations in continuous dynamical systems. Their account hinges on the observation that the dynamics on a network defines a global attractor that dictates the curvature of the phase space [290], compatibly with the metastable brain framework. Specifically, sets of solutions and the connections between them (e.g., heteroclinic channels) define the system’s informational structure; the attraction rates of each state define an informational field [290]. Those can then be used to derive state transition probabilities [290]. In agreement with this continuous variant of IIT, we hold that the representational structure of conscious neural systems—the set of metastable states and the transition probabilities between them—must be well-defined for any choice of (finite) spatiotemporal scale. Future research may, using such constructs (perhaps in combination with the methods described in Section 2.3), relate quantitative descriptions of spatial and object-based attention to mechanistic accounts invoking pulvinocortical oscillatory dynamics.

### 4.3. Inference through Gradient Descent in the State Space

Compatibly with the metastable brain framework, Friston [53,291] proposed that self-organized [21,79] biological systems resist a tendency to disorder, have relatively low entropy, and only ever visit a small portion of their state space. The free energy in a neural system is defined as an upper bound on the negative log-probability of observed inputs; sensory processing, on this view, can be thought of as gradient descent on this quantity [53,292]. Friston [293] defines causes as processes that perturb the system in question (defined as a set of differential equations); in nature, those are mutually dependent and often hierarchical. He posits that the brain learns a generative model of its inputs and their causes and uses it to estimate the probability of putative causes conditional on the input; the observed quantities are nearly always a nonlinear function of multiple unobserved latent variables [293]. 

Ongoing neural activity, on this account, dynamically encodes a probability density function of hidden causes given the current brain state [53,293]. Minimizing prediction error online reduces to minimizing the sum of the negative log probability of events given the learned parameters and the Kullback–Leibler (KL) divergence between the recognition density and posterior density (i.e., free energy) [291]. Thus, following a perturbation, neuronal dynamics should (with everything else kept constant) tend to a state that encodes an optimal guess as to the causes of the input, as the KL divergence tends to zero [53]. Under realistic conditions, however, this process would be interrupted by subsequent perturbations external to the ensemble in question [294]. Neural state transitions can thus be thought of as a gradient flow on variational free energy. They are dictated by the geometry of the state-space manifold [84], which in turn is shaped by synaptic learning. Spike timing dependent plasticity [194], for instance, would entail that repetitive spike patterns should make information more likely to be routed to previously-traversed pathways—a dynamic that may facilitate statistical learning, inference, memory, and attention [172]. 

Generative brains of the sort envisioned by Friston and colleagues [295] may exhibit winnerless competition or chaotic itinerancy—the former may correspond to more controlled state transitions (e.g., due to signals from the frontoparietal attention network to lower-level sensory areas), whereas the latter may subserve non-serial searches (e.g., in creative tasks such as the remote associates test [296]) [52,93,94]. In both cases, the transition probabilities given a particular connectivity matrix would reflect statistical regularities that existed within and between previously maintained states.

## 5. Conclusions

To conclude this article on a forward-looking note, we would like to touch upon a more fundamental question: what mathematical descriptions would afford the most complete account of representational dynamics in neural systems? Field models [72,73,74,75,297] seem an apt choice, seeing as informative patterns of neural excitation (be they carried by spikes, local field oscillations, or both in a nested fashion) correspond, by definition, to structured fluctuations in the electromagnetic field [136]. A renewed appreciation of this fact has resulted in a recent surge of interest in neural mean-field models [101], constructed and tuned based on connectivity data [34,298,299]. There is clear evidence tying such formal descriptions of field potential dynamics to those underlying visual phenomena (from binocular rivalry [106] to patterned visual hallucinations [105]) [34]. The oscillatory patterns observed in human electrophysiological data have been described as “quasi-stable… phase structures consistent with global standing waves” [300] (p. 125)—one form in which metastable neural dynamics could manifest in an underlying physical field [34,136]. Recent insights into the oscillatory dynamics of the primate visual system could galvanize efforts to relate spike- and LFP-level descriptions to wave packet formalizations.

The advent of models, theories, and analysis methods that consider the geometry and topology of spatiotemporally extended neuronal activity patterns is a welcome development in cognitive neuroscience. When carefully matched with empirical data, dynamical models can provide useful predictions. The dynamical approach also carries with it the promise of fostering integration between neuroscience, physics, and other nonlinear sciences: as we learn more about the mechanisms and laws underpinning long-range coordination in thalamocortical networks, the principles underlying the behavior of other complex systems may also be illuminated [125].
